# Green Leaf Volatiles: A New Player in the Protection against Abiotic Stresses?

**DOI:** 10.3390/ijms25179471

**Published:** 2024-08-30

**Authors:** Jurgen Engelberth

**Affiliations:** Department of Integrative Biology, The University of Texas at San Antonio, San Antonio, TX 78247, USA; jurgen.engelberth@utsa.edu; Tel.: +1-210-458-7831

**Keywords:** abiotic stress, airborne signal, green leaf volatiles, GLV, plant damage, plant protection, structural integrity, volatile organic compounds

## Abstract

To date, the role of green leaf volatiles (GLVs) has been mainly constrained to protecting plants against pests and pathogens. However, increasing evidence suggests that among the stresses that can significantly harm plants, GLVs can also provide significant protection against heat, cold, drought, light, and salinity stress. But while the molecular basis for this protection is still largely unknown, it seems obvious that a common theme in the way GLVs work is that most, if not all, of these stresses are associated with physical damage to the plants, which, in turn, is the major event responsible for the production of GLVs. Here, I summarize the current state of knowledge on GLVs and abiotic stresses and provide a model explaining the multifunctionality of these compounds.

## 1. Introduction

Green leaf volatiles (GLVs) are a group of plant compounds that are typically associated with damage. Most people have experienced these molecules when mowing their lawns and recognize them as the typical “green” smell of plants. For more than 20 years now, GLVs have come to our attention as volatile signals within and between plants that communicate damage, usually caused by insect herbivores, but also by microbial infections [1]. In doing so, GLVs have been found to not only provide immediate protection by activating defensive measures, but also to prepare or prime receiver plants against the threat of impending damage [2,3]. Generally, priming may initially trigger only a minor part of a defense response, which then leads to an increase in the plant’s ability to defend itself against future antagonists (for example, herbivores or pathogens), resulting in a faster, stronger, or more enduring response when actually being attacked [4]. Fittingly, GLVs have their own defense-related biological activity, which is, however, considered to be rather weak when compared to responses signaled, for example, by jasmonic acid, which is the major defense hormone that regulates responses to herbivory and necrotrophic pathogens. Nonetheless, defense priming by GLVs appears to be strongly connected to the jasmonate pathway in that signaling through it becomes more intense [2,3]. Still, little is known about how defense priming actually works. It seems clear that some memory is conserved after the first exposure to a priming agent, including the accumulation of mitogen-activated protein kinases (MAPKs) that remain inactive until triggered by a threat, or epigenetic changes [4]. However, with regard to priming by GLVs, no such mechanisms have been reported with sufficient evidence to accept them as possible regulators of priming. 

While most reports on GLVs in the past have focused on their role in mediating biotic interactions, GLVs have more recently been found to provide protection to receiver plants against abiotic stresses, either directly or by priming for them. This includes protection against various stresses, including cold, drought, salt, and light. Still, very little is known about the mechanisms by which GLVs act in mediating abiotic stress protection. In this review, I will therefore summarize GLV-induced protective activities related to abiotic stresses that are signaled by this group of compounds and provide some insight into the potential mechanisms by which they may achieve this. 

## 2. Green Leaf Volatiles and Abiotic Stress

### 2.1. The Biosynthesis of Green Leaf Volatiles

**Figure 1:**
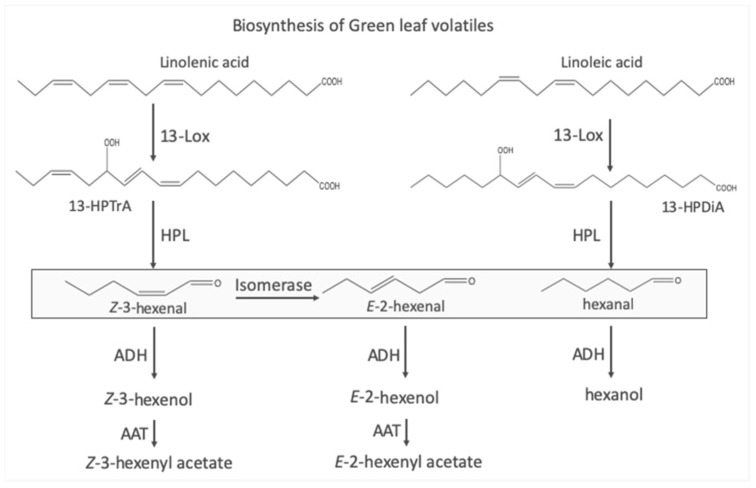
Biosynthetic pathways leading to the production of green leaf volatiles (GLVs) (taken from [11]). A lipoxygenase catalyzes the addition of molecular oxygen at position 13 in linolenic acid or linoleic acid, resulting in 13-hydroperoxy octadecatrienoic (13-HPTrA) or 13-hydroperoxy octadecadienoic acid (13-HPDiA). The oxygenated fatty acids are then cleaved by a hydroperoxide lyase (HPL) into either Z-3-hexenal or hexanal. The remaining 12-cabon unit is further processed into traumatin (not shown). An isomerase can convert Z-3-hexenal into E-2-hexenal. Both aldehydes can be further processed by an alcohol dehydrogenase (ADH), resulting in the respective alcohol. Further modifications by an alcohol acyltransferase (AAT) can convert the alcohols into the corresponding hexenyl acetates. The boxed compounds are those GLVs produced mainly by damaged plant tissue, while alcohols and their esters require intact cells for biosynthesis.

The biosynthesis of GLVs is rather straight-forward, starting mainly with linolenic acid, either in its free form or as part of typical membrane lipids (Figure 1) [5,6]. In the first step, a lipoxygenase (LOX) inserts molecular oxygen at position 13 of the fatty acid. A hydroperoxide lyase (HPL) then cleaves the fatty acid into a 6-carbon compound, Z-3-hexenal (Z3al), and a 12-carbon unit, which, after a minor conversion, results in traumatin, a molecule that has been recognized as a wound hormone capable of inducing callus formation [7]. The six-carbon unit Z3al is then reduced to its corresponding alcohol (Z-3-hexenol (Z3ol)), which can then be further modified into various esters, mostly into Z-3-hexenyl acetate (Z3ac). Additionally, some plants also have an isomerase that can quickly convert Z3al into E-2-hexenal (E2al) [8,9], which can also be transformed into the corresponding E-2- alcohol and esters. While LOX and HPL are commonly localized in chloroplasts, all other enzymes, including the isomerase, are cytoplasmic. However, upon damage, LOX and HPL become activated, resulting in the rapid production of Z3al. If cells contain an isomerase, it will also become highly active in the damaged tissue and almost instantly transforms Z3al into E2al. In contrast to these initial steps of GLV biosynthesis, all other reactions require intact cells, which take up the aldehydes and transform them into the corresponding alcohols and esters [10]. 

Plants can produce significant quantities of GLVs within seconds to minutes after damage, some up to almost 100 µg per gram of fresh weight [1,11]. This substantial and rapid production makes them ideal volatile signaling molecules for either distant parts of the same plant or other plants in the vicinity. While little is known about how exactly Z3al is made in damaged tissue and what regulates the process, we found that even at temperatures far below 0 °C, damaged plant tissues can still produce significant amounts of Z3al under those conditions [12], while the alcohols and esters are barely detectable, mainly because most cells under these conditions have died and can no longer produce these compounds. However, while basically all plants produce GLVs in various quantities and qualities upon damage and other treatments, most experiments to date have been conducted by using pure chemicals that are commercially available, which allows for a more controlled application of these volatile compounds. The experimental risk here is that the concentrations used may not correlate with what can be found in nature and may produce artifacts. Often, nano- to low micromolar concentrations have been found to sufficiently signal GLV activities. Yet, plants may also experience much higher concentrations upon damage, particularly in the immediate vicinity of the damaged tissue [2,10]. It is therefore often impossible to assess the biological activities of these compounds in contexts with high concentrations being experienced locally but low concentrations serving as a volatile signal over relatively long distances. 

Nonetheless, signaling pathways related to GLV activities are currently being elucidated at various levels. Exposure to GLVs, for example, can cause rapid changes in membrane potentials and cytosolic Ca^2+^ concentrations [13,14]. It has been argued that GLVs themselves cause this depolarization directly due to their hydrophobic character and potential interaction with membranes. However, the distinct set of genes that are induced by GLVs and the specificity of the primed responses argues for a more regulated signaling pathway. However, since no receptor has been identified to date, neither mechanism can be excluded. 

It further appears that Z3ol is the major biologically active form of GLVs [15]. In a series of experiments performed by Cofer et al., it was shown that after mutating several enzymes involved in the hydrolyzation of GLV esters like Z3ac, the overall activity of these compounds was dramatically reduced, clearly pointing towards Z3ol as the main active molecule. Furthermore, certain GLVs seem to activate a MAP kinase signaling pathway in tomato plants [16]. Interestingly, the same MAP kinase pathway is normally used after pathogen infection. In support of these findings, we found in a microarray study that determined that one MAP kinase was significantly induced in maize plants treated with Z3ol, suggesting that it might be a GLV-specific response [17]. Yet, it is still unclear if this is a common signaling pathway that is recruited by GLV or what other signaling pathways might be involved in regulating responses to these compounds. The activation of the MAP kinase pathway could, however, provide a link to the priming responses and the associated memory effects, as described above [4]; however, this is a hypothesis that still needs to be tested. 

### 2.2. Green Leaf Volatiles in the Atmosphere

As mentioned before, the bioactive roles of GLVs have mostly been studied in the context of plant–insect and plant–pathogen interactions, both of which were also shown to cause the release of significant quantities of GLVs not only from the damaged tissues, but also from undamaged parts of the same plant [1,2,18]. It was further found that the treatment of plants with GLVs often prepared or primed them against the impending threat, resulting in a stronger and/or faster response when actually attacked [2,3,19,20,21,22]. This led to the assumption that GLVs mainly have a role in the defense against biotic threats. However, in recent years, several reports have shown the potential for GLVs also being involved in regulating abiotic stresses. Initial reports came from studies investigating the composition of volatile organic compounds, which comprise all volatiles regardless of their origin, in the lower atmosphere of the Earth. There, large quantities of so-called biogenic compounds (compounds that are emitted from organisms) were detected and their effects on the chemistry of the atmosphere studied [23,24]. While the majority of these biogenic volatile compounds were found to be isoprene-related, which are usually emitted in large quantities under heat and light stress, where they provide cooling as well as helping in the recovery of photosynthetic performance, significant amounts of GLVs were also detected. Since it is unlikely that herbivory or pathogen infections are the sole cause of the presence of GLVs in the atmosphere, other factors like grass harvesting for hay in agriculture as well as abiotic stresses need to be taken into consideration. Proof of the latter came, for example, from studies by Karl et al. [25] and Jardine et al. [26]. Karl et al. [25] found increased GLV levels in frost-damaged meadows in the alps, with Z3al being the major compound. Jardine et al. [26] analyzed the composition of the air surrounding the canopy region of rainforests in South America. The authors not only detected large quantities of GLVs, but also found a clear correlation between the amount of GLVs and abiotic stresses like drought and heat, and concluded that atmospheric GLVs could be used as a chemical stress sensor. Similar results were provided by Turan et al. [27] when they investigated the effects of heat on tobacco leaves. When plants were exposed to 52 °C, they produced large quantities of E2al and Z3ol, but very few terpenes. This production of GLVs under heat stress indicates that some kind of membrane damage or at least disturbance occurs resulting in the activation of enzymes in their biosynthetic pathway. Since Z3ol was among the detected compounds, it can also be concluded that even at those high temperatures, intact cells are still abundant and can convert aldehydes into the alcohols. Based on these clear correlations between abiotic stresses and GLV release, it can be assumed that there should also be a functional connection. However, at the time, little was known about how GLVs might contribute to protection against these stresses. 

### 2.3. Green Leaf Volatiles and Cold Stress

Cold stress poses a serious threat to plants. While it is often a seasonal issue, it can still affect plants in regions closer to the poles or at higher altitudes, even during the summer. However, the highest risk of plants experiencing cold stress usually occurs during spring time in temperate climates, when they germinate, and in the fall, when they are usually harvested. Cold stress can cause significant changes in the general physiology of plants. Cold stress may generally reduce enzyme activities, can cause cell membrane damage through altered membrane fluidity and lipid composition, decreases water potential, reduced ATP supply, and may bring about imbalanced ion distribution and solute leakage (Theocharis et al. [28]). This does often result in reduced growth and yield. It is therefore important for plants to have mechanisms in place that help to prevent severe consequences of this stress. 

Karl et al. [25], as described above, provided the first insight that GLVs might be a part of such a strategy, since cold-damaged plants emitted significant quantities of these compounds. Likewise, Copolovici et al. [29] found that cold stress caused the release of large quantities of GLVs in cold-stressed, but also in heat-stressed, tomato (*Solanum lycopersicum*) plants. Similar to Jardine et al. [26], the authors proposed the use of these volatile organic compounds as indicators to characterize the severity of the stress. Yet again, no studies have been performed that would test for a protective role of GLVs under these conditions.

In 2013, we published a microarray study on the effects of Z3ol, in particular, on general gene expression in maize [17]. We focused on early events and identified distinct expression patterns, many of which were likely related to defense reactions to insect herbivores. Unfortunately, at the time, many genes on the microarray were still unknown or mislabeled (about 40%). Later, we identified several genes that were typically associated with water stress in plants, including dehydrins, low-temperature-inducible protein, and several others [30]. This pointed towards a potential role of GLVs in protecting cellular integrity, since these types of proteins are usually involved in stabilizing cell structures, including membranes. Upon further analyzing the effects of GLVs on cold stress protection, we found not only that the expression of the identified protective genes was induced by GLVs, but also that their expression levels were primed when they were placed in the cold about 2 h after GLV treatment. This resulted in significantly reduced ion leakage [12,31], less damage, and a growth spurt in the days after the cold stress treatment. Aside from providing immediate protection, this also indicated an effect of GLVs on the general physiology of the plant, which allowed it to compensate for a loss of growth during cold stress. We also found that maize plants do still produce GLVs, in particular, Z3al, at temperatures well below 0 °C [12]. Furthermore, Z3al was able to increase the transcript accumulation of those protective genes even when applied during cold stress [12]. This proved that GLVs can protect plants against cold stress even when perceived during a cold episode and also provides a potential mechanism by which this might be achieved, i.e., the activation of cell-protective proteins, and thus, the maintenance of cellular integrity. This is essential since it allows cells to continue to function properly even under potentially damaging stress conditions. 

### 2.4. Green Leaf Volatiles and Drought Stress

Drought is defined as an absence of water (https://www.ncei.noaa.gov/access/monitoring/dyk/drought-definition (accessed on 28 August 2024)). However, an absence or shortage of water can also be the result of high salt concentrations or an abundance of other water-capturing chemicals, all of which result in a dramatic change in the water potential of plants. While the early effects of drought and salt stress are very similar, both stresses act through distinct signaling pathways. Additionally, salt may also have a toxic ion effect and can lead to nutritional imbalances in plants [32,33]. This makes it more difficult for the plant to maintain a constant transpirational stream, which can lead to the overheating of leaves, but also to the reduced uptake of nutrients. Consequently, plants exposed to drought grow less and provide much lower yields. Water scarcity is therefore one of the most pressing issues when growing plants in a natural environment or in an agricultural setting. 

The potential role of GLVs in drought stress responses was initially provided by Jardine et al. [26] through their analysis of the atmosphere of the forest canopy in the rainforest, in which they found a clear correlation between drought, temperature, and GLVs. This was the first clear indication that GLVs may play a role in the regulation of drought and heat stress in a natural system. However, at the time, it was unclear if there might also be a protective role that GLVs play in this context, since studies in a natural system are difficult to perform due to the myriad of other environmental factors that may interfere. In a related study by Catola et al. [34], it was further shown that drought stress affects the capacity to produce GLVs in the leaves of the pomegranate plant (*Punica granatum* L.), further supporting the involvement of GLVs in the response to drought stress. Yet again, no further studies have been performed to evaluate the potential protective role of GLVs.

However, evidence for GLVs playing an active part in protection against drought-related stresses came from a study by Yamauchi et al. [35]. By investigating the potential for activating gene expression, they tested an array of reactive leaf volatiles in a microarray assay on Arabidopsis. While the focus of the study was on reactive α, β-unsaturated carbonyls, they identified E2al as a particularly effective inducer of typical abiotic stress-related genes, including those that protect against drought and salt, but also heat and cold. At the same time, Z3al appeared to be quite inactive and did not show any significant induction of abiotic stress-related gene expression, which could, however, be a species-specific result. While this did not answer the question of whether or not GLVs do actually provide protection against drought, and salt stress in particular, a study by Tian et al. [36] showed that priming with Z-3-hexenyl acetate enhanced salinity stress tolerance in peanut plants (*Arachis hypogaea* L.). As a result, they found positive effects on photosynthesis, higher water content, increased growth, and increased activity of antioxidant proteins. While this broad spectrum of protectionist measures may be surprising, it actually fits into the overall picture of activities provided by GLVs that have been shown for biotic stress responses [3]. 

A similar study investigated the effects of Z3ol on hyperosmotic stress tolerance in *Camellia sinensis* [37]. As described above, a multitude of effects were found, ranging from regulating stomatal conductance, decreasing malonyl dialdehyde as an indicator of lipid peroxidation, the accumulation of abscisic acid and proline, and typical stress-related gene expression. The activation of ABA and proline, in particular, is interesting, since both are essential responses to hyperosmotic stress: ABA by acting as the major regulator of water stresses [38], and proline by functioning as an important protector of cellular integrity [39]. The involvement of ABA as a mediator of Z3ol-induced protection against drought and cold was further confirmed by Jin et al. [40] in *Camellia sinensis*. They showed that Z3ol activated the glycosylation of ABA through the expression of a specific glycosyl transferase. This allows ABA–glucose conjugates to be stored in the vacuole, from where they can be easily reactivated upon cold and drought stress by a glucosidase. While this provides an elegant system that helps to explain some of the biological activities of GLVs (here: Z3ol), it still needs to be confirmed in other plants. Furthermore, while Z3ol was shown to be the active compound in this study, other GLVs—in particular, Z3al, as the one compound that is instantly produced by damage, including cold—also need to be tested for their specific activity towards the activation of ABA-mediated signaling as a key element in this process. 

Altogether, these results clearly show that GLVs are not only released upon drought stress in significant quantities, but can also provide significant protection. Furthermore, these experimental results provide a potential mechanism by which GLVs may activate these processes with ABA, the major regulatory plant hormone for water stresses, being a central target.

### 2.5. Green Leaf Volatiles and Photosynthesis

Light is a determining factor in the life of plants. It is the predominant energy source that is used by plants and other photosynthetically active organisms, which transform light from a physical power into energy-rich molecules that are essential for the vast majority of living things on Earth. For plants, light also serves as a signal that has a significant impact on growth as plants make an effort to obtain a perfect position for light harvesting. However, light can also be too powerful and under these conditions, and plants may sustain damage due to the high energy levels that are contained in the radiation. This is, among other consequences, extremely challenging for the actual photosynthesis reaction, in particular, the events that occur in photosystem II (PSII). There, the photolysis of water represents one of the main events during photosynthesis, resulting in the production of electrons, protons, and eventually oxygen. Under normal light conditions this is a well-controlled process. But when light intensities increase, oxygen radicals are produced, which can cause significant harm to the whole photosystem, but also other parts of the chloroplast. And it is this process in which GLVs appear to interfere by regulating the status of PSII in particular. 

First indications to support a role of GLVs in photosynthesis came from a study by Charron et al. [41]. They found that an increase in the photosynthetic photon flux and the length of the photoperiod would cause an increase in GLV production and release in lettuce. While this was mainly put in the context of growing plants in controlled environments, it nonetheless pointed towards GLVs being directly linked to light stress. Further proof came from a study on bacterial photosynthesis. Mimuro et al. [42] investigated the effects of 1-hexanol on the optical properties of the base plate and energy transfer in *Chloroflexus aurantiacus*. In this system, it was found that adding 1-hexanol caused the suppressed flux of energy from the baseplate of the chlorosome to the photosynthetic elements located in the adhering plasma membrane section. While it is still unclear whether or not bacteria can produce GLVs, the results nonetheless provided evidence that these compounds can have an effect on photosynthetic reactions. Furthermore, considering that this kind of photosynthetic microbe might represent an ancestorial type of what may have eventually ended up as a chloroplast in plants, mechanisms to protect or at least alter the system may also have been already abundant. 

Negative alterations of photosynthesis were further investigated by Matsui et al. [10]. While studying the metabolism of GLVs, they identified key mechanisms of the biochemical pathway. As described above, aldehydes are mainly produced in damaged tissue, whereas the corresponding alcohols and esters require intact cells to be made. Aside from identifying these mechanisms, they further investigated the toxicity of various GLVs, including Z3al, E2al, and Z3ol, and found that plants exposed to the compounds as pure chemicals showed a significant impact on photosynthesis, with the aldehydes being much more active than the corresponding alcohol. Similar results were obtained by Tanaka et al. [43]. Together, this led to the conclusion that plant cells have an intrinsic ability to detoxify the much more reactive aldehyde GLVs into less harmful alcohols and esters, which, in turn, appear to serve as the actual signaling molecules that regulate all processes attributed to GLVs [15]. At the same time, plants avoid the toxic effects of the aldehydes. However, new light was shed on this issue when Savchenko et al. [44] studied the effect of GLVs on photosynthesis. By using HPL-overexpressing lines in Arabidopsis, they discovered that GLVs play a major role in the protection of PSII under increased light conditions by observing lower rate constants of PSII photoinhibition and higher rate constants of recovery. Furthermore, the degradation of proteins (in particular, D1, but also others) during photoinhibition was significantly reduced, allowing for a more speedy recovery. Further experiments on isolated thylakoid membranes confirmed these results. In contrast to GLVs, the application of other oxylipins, including linolenic acid, phytodienoic acid, and jasmonates, had the opposite effect, further solidifying the specificity of GLVs’ effects on PSII. This important feature of GLVs may also explain why this biochemical system is localized in chloroplasts, more specifically, in the thylakoids. Reducing the uncontrolled production of reactive oxygen species (ROS) may be key in the protection of plant cells under extreme light conditions and may explain why GLVs have a negative impact on photosynthesis, which, while considered toxic in the past, actually help to avoid damage in leaf cells. 

However, little is known about the actual mechanisms that are activated by GLVs to protect against light stress. Further studies in the area of light stress protection in particular are needed to identify these mechanisms, which may, in turn, further help to explain the diverse and complex roles GLVs play in plants. 

## 3. Future Perspectives

**Figure 2:**
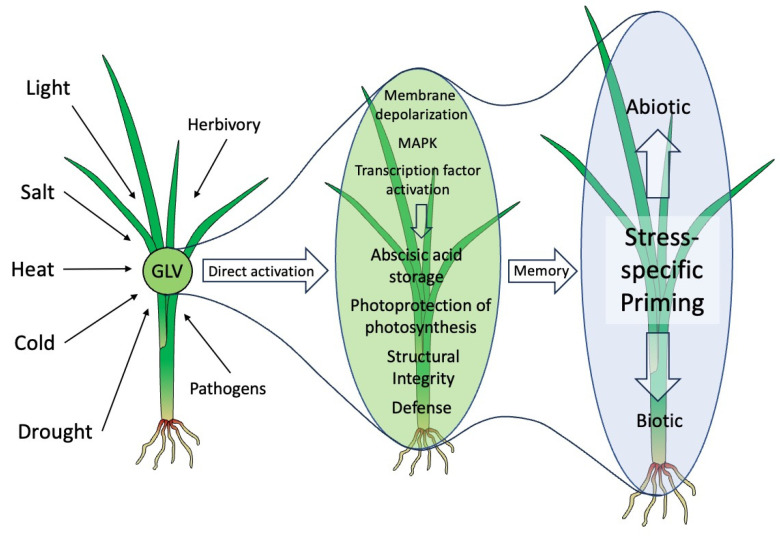
Green leaf volatiles and abiotic stress responses. Green leaf volatiles (GLV) are produced by many plants under various abiotic stress conditions. As volatiles, they move as a plume through the air and can reach neighboring plants, where they can activate various protective measures directly through specific signaling pathways, resulting in abscisic acid storage, the photoprotection of photosynthesis, increased structural integrity, and defense activation. Aside from these more direct measures, GLVs may prepare or prime plants against impending stresses through a yet-to-be-identified memory effect, which allows them to respond faster and/or more strongly when actually threatened. These primed responses are specific towards the actual threat and are likely a consequence of the direct activation of protective measures by GLVs. The direction of the activation from left to right in this figure represents a time scale and not the distance between plants. The green background shows more direct effects, while the blue background represents priming.

The definition of GLVs as a plant’s multifunctional weapon [3] was created at a time when mainly its defensive functions were recognized. This view has now been expanded by adding a multitude of abiotic stresses that are also covered by GLVs, as described herein. A summary of these findings is presented in Figure 2.

This extreme multifunctionality raises the question of how these compounds can be utilized to better plant protection against biotic and abiotic stresses. A summary of approaches towards protection against biotic stresses by volatile organic compounds in general was provided by Wang et al. [45]. Two major paths were outlined, one using intercropping with sentinel plants, and the other using pure chemicals to help to protect plants. These approaches could also be chosen for using GLVs as protectors against abiotic stresses. Intercropping would require a sentinel plant that is more sensitive to certain abiotic stresses, in particular, drought and cold, and that produces GLVs upon being exposed to these stresses in sufficient quantities to protect the main crop. At the same time, these sentinel plants should not take away too many of the nutrients and, ideally, should continue to grow even after suffering damage to allow for longer-term protection. Alternatively, pure chemicals like Z3ol or Z3ac could be deployed over fields at critical times to assist in protection against relevant abiotic stresses. However, there are several issues here that need to be addressed. One is costs, which could be significant. Another issue lies in the fact that these compounds are volatile and may dissipate very quickly, making it necessary to repeat the application regularly. At this time, we do not know how long a protective or priming effect against certain stresses remains active within a certain plant species, but this is necessary information that would determine how often such a treatment would have to be repeated to be effective. One solution could be the development of a slow-release mechanism for GLVs or to produce a conjugate that can be taken up by the plant without dissipating as a volatile into the atmosphere. Another issue is that while GLVs can protect against a variety of biotic stresses, they may very well interfere with alternative defense signaling pathways. As mentioned before, GLVs appear to act through the jasmonate signaling pathway, and in doing so, protect against many insect herbivores and necrotrophic pathogens. However, this does, for example, allow biotrophic pathogens to infect plants and thrive. For biotrophic pathogens, salicylic acid is the major defense regulator. However, its activity is down-regulated by the jasmonate pathway and vice versa [46]. These interactions need to be fully understood before treatments that activate one or the other pathway can be deployed. It also necessitates more studies on how GLVs may interfere with other signaling pathways to avoid any negative side effects with significant impacts on growth and yield. To date, no such study has been performed in the area of GLVs and abiotic stress responses. 

Another path forward could lie in the molecular manipulation of GLV production and sensing. While we know most of the genes involved in their biosynthesis [5,6,7,8,9], we are still at the beginning of understanding how GLVs are sensed. But even with biosynthesis, we only have a limited understanding of the regulation of the initial steps, in particular, after tissue damage, but also regarding the release of GLVs from intact tissues. In this context, an interesting finding is the release of a burst of GLVs at the onset of darkness. This has been described for some plant species [47,48], but it is yet unknown why plants do this. Also, this GLV burst seems to require a rather quick transition from light to dark. However, exploring and eventually exploiting this phenomenon of potentially self-priming may provide a starting point for future manipulations, resulting in the better protection of plants. 

We still know very little about how plants perceive GLVs as signals and exactly how they transduce them into a physiological response. Is it just the physical perturbation of membranes, or are specific receptors involved? These are questions for which we do not have any answers at this time. Once resolved, this information could provide more targeted approaches for manipulation. Increasing the sensitivity of plants to GLVs might be one promising line of exploitation. Overexpressing key enzymes might be one way, but also, providing the substrates for the reaction (i.e., poly-unsaturated fatty acids) may help to boost the production of these compounds. However, as mentioned above, manipulating one signaling pathway may have severe consequences for others, and this must therefore be carefully studied with consideration of the whole plant’s physiology. For example, only a few studies so far have looked into the costs of GLV-activated responses [49,50,51]. Surprisingly, the application of these compounds has often resulted in an increase in growth [12,31,49,50,51]. Should this be confirmed for other plant species and stresses, it might very well justify more thorough investigations into the feasibility of using GLVs as an almost universal protection system that would not have negative effects on other physiological responses. 

However, our knowledge regarding GLV regulation is still very limited, not only with regard to the enzymes involved, but also when it comes to how different plant species regulate the process of producing and sensing these compounds. This becomes extremely clear when we look at the number of plants that have been used to provide the results presented herein. Aside from Arabidopsis, fewer than 10 different plant species have been used to study the effects of GLVs on abiotic stress protection. Furthermore, the experimental approaches are very different depending on the goal of the study. I believe that a more comprehensive and coordinated approach is indeed necessary to better understand how GLVs affect plants on a global scale. A summary of current findings regarding the protective roles of GLVs against abiotic stresses is shown in Table 1.

## 4. Summary and Conclusions

While the number of publications on the topic of GLVs and abiotic stress protection is far exceeded by those on GLVs and biotic stresses, it cannot be denied that GLVs can provide significant protection through yet-to-be-identified signaling pathways. But even with the limited number of publications on the topic, it is astounding how GLVs can protect against such a broad variety of abiotic stresses. If we compare this with other plant signaling compounds, including hormones like jasmonic acid, abscisic acid, salicylic acid, and many others, it becomes obvious how limited these are in the regulation of protective functions when compared to GLVs. This broad protection makes GLVs somewhat unique within the regulatory network of plant stress responses.

To best describe the protective roles of GLVs, one has to look at what is causing damage to plants. Damage in most instances is physical, and thus directly linked to water loss, which may lay at the core of GLV activity. But it clearly goes beyond that when they also protect against herbivores, pathogens, drought, cold, intense light, salt, and other damaging stresses. Also, while they protect against those stresses, they are also often released by the same stresses they protect against, thereby potentially providing protection to other parts of the same plant or even other plants nearby, either by directly activating protective responses or by priming for them. This will result in a faster and/or stronger reaction should the stress actually occur. Surprisingly, all this is carried out with minor investments, making this a very low-cost investment on the plant’s side. 

GLVs have been labeled ‘the plants multifunctional weapon” in the past based on their multifaceted biological activities against biotic stresses in particular [3]. It is, however, obvious from the findings summarized herein that we have to expand this characterization to include protection against abiotic stresses as well. I would therefore also not describe GLVs as new players in this context, but rather, as very old ones that just need to be further studied to reveal their full potential in regulating a multitude of stress responses in plants. 

To conclude, the role of GLVs seems to lie in in the protection of plants against all those stresses, biotic and abiotic, that can cause damage in the widest meaning of this word. However, how these complex activities are regulated by these compounds is mostly still unknown. But the potential of GLVs in protecting plants on such a broad scale is definitely worthy of further investigation.

## Figures and Tables

**Table 1:** Protective effects of green leaf volatiles in different plant species.

Stress	Organism	Active Compound	Responses	Citation
Cold stress	*Zea mays*	Z-3-hexenal Z-3-hexenol	Abiotic stress genes Reduced ion leakage Reduced damage	[12,31]
Drought	*Arabidopsis thaliana*	E-2-hexenal	Abiotic stress genes	[35]
	*Arachis hypogaea*	Z-3-hexenyl acetate	Photosynthesis Increased water content Growth Antioxidant proteins	[36]
	*Camellia sinensis*	Z-3-hexenol	Stomatal conductance Reduced lipid peroxidation ABA accumulation Proline accumulation Stress gene expression	[37]
	*Camellia sinensis*	Z-3-hexenol	ABA glycosyl transferase ABA storage	[40]
Photosynthesis	*Chloroflexus auratiacus*	1-hexanal	Reduced energy flux	[42]
	*Arabidopsis thaliana*	E-2-hexenal Z-3-hexenal E-2-hexenol	Reduction in photosynthetic activity	[10,43]
	*Arabidopsis thaliana*	E-2-hexenal Z-3-hexenal Z-3-hexenyl acetate	Protection of PSII Recovery Reduced protein degradation	[44]

## Data Availability

Not applicable.
